# Incorporating Animal Social Context in Ecotoxicology: Can a Single Individual Tell the Collective Story?

**DOI:** 10.1021/acs.est.1c04528

**Published:** 2021-07-28

**Authors:** Jake M. Martin, Erin S. McCallum

**Affiliations:** † School of Biological Sciences, Monash University, 3800 Melbourne, Victoria Australia; ‡ Department of Wildlife, Fish & Environmental Studies, Swedish University for Agricultural Sciences, 90183 Umeå, Sweden

**Keywords:** chemical pollution, social environment, social behavior, group behavior, adverse outcome pathway

**Figure 1:**
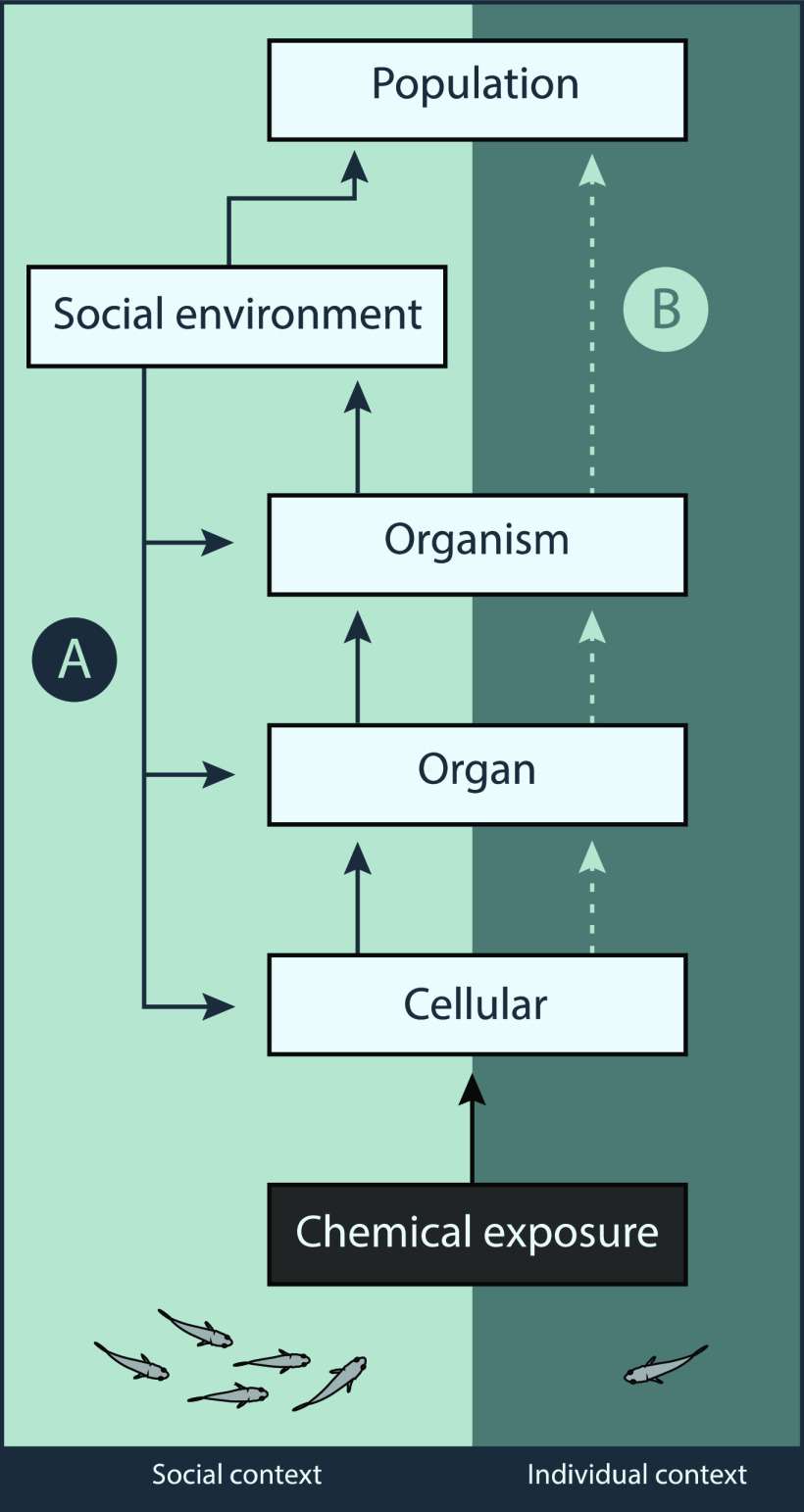
Theoretical representation of the role the social environment can play in a typical adverse outcome pathway (AOP) framework. Illustrating (A) the potential for an organism’s social environment to create bidirectional feedback at different levels of the AOP (specific examples illustrated in text), and thus through its inclusion, can increase predictive power along the AOP (represented by solid arrows). This is contrasted by (B), an AOP which uses data from an individual context alone to predict population level outcomes, and thus has less predictive power along the AOP (represented by dashed lines).

Chemical pollution is an insidious and growing threat to ecosystems globally. Over five thousand different chemicals are regularly detected in the environment, and less than half have undergone any safety or toxicity assessment. The overarching goal of ecotoxicology is to detect and predict the impacts of these contaminants on the natural world. To do this, researchers often employ experiments that simplify and compartmentalize the natural world. Data from these experiments are then used to identify adverse outcomes, with the aim to extrapolate laboratory findings to a real-world setting. Inherently, this process contains assumptions and generates uncertainties. One widespread assumption is that the impact(s) of a contaminant on an organism in a social void—that is, exposed, tested, or housed in isolation—is predictive of the impacts seen in a social environment. For us, this is a surprising assumption because elements of an organism’s social environment are likely to mediate the impacts of contaminants and could do so at multiple levels of biological organization (or the adverse outcome pathway; Figure 1). Moreover, through relatively minor changes in common methodologies, this assumption can be mitigated or sidestepped altogether. In this viewpoint, we will illustrate (1) why the social environment is important in the context of ecotoxicology, (2) how it might mediate chemical impacts at multiple points along an adverse outcome pathway, and (3) barriers to incorporating a social context in ecotoxicology and we recommend solutions.

Here, we refer to the social environment as the context in which social interactions occur, including the characteristics of the group, the features of the surrounding environment, and the social interactions themselves (e.g., mutualistic, commensalistic, and antagonistic interactions). In natural ecosystems, most species spend at least part of their lives in some form of social environment, whether in transient aggregations (e.g., to reduce predation risk) or more stable long-term groups (e.g., with complex hierarchical and competitive structures). It is well-known that an organism’s social environment can influence their physiological state (e.g., neuroendocrine signaling, metabolism) and behavioral expression (e.g., foraging, aggression, mating), which in turn, can affect end points important in ecotoxicology like growth, reproduction, and survival.^1^

Chemical exposures can alter an animal’s social environment by disrupting their responsiveness to social cues and/or their ability to perceive social cues. For example, exposure to copper nanoparticles can impair olfactory neural signals, reducing the perception of conspecific cues in rainbow trout (*Oncorhynchus mykiss;*^2^). The impacts of such chemicals would, therefore, only be realized under a social context and would not manifest (or manifest differently) if tested in a nonsocial setting. Our own recent work provides direct examples of this, where the effects of chemical exposure (the pharmaceuticals fluoxetine or oxazepam) on the growth and foraging dynamics of fish was mediated by the social environment—the presence of conspecific group members or position in social hierarchy.^3,4^ Chemical disruption of the social environment can also have consequences beyond individuals and their immediate group. For example, in a competitive reproductive environment (e.g., dominance hierarchies), a chemical exposure that changes social phenotypes underlying which individuals successfully reproduce could shift paternity and ultimately change the selective regime the population experiences (e.g.,^5^).

Above, we highlight several examples of how the impacts of a chemical, if tested in isolation, may not be predictive of impacts in a more natural social environment. Thus, the absence of a social environment could introduce uncertainty along multiple points of the adverse outcome pathway (Figure 1). Yet despite this, the social environment of study species is ***not*** widely incorporated into modern ecotoxicology research (with some taxa being notable exceptions; e.g., Hymenoptera). We surmise that this is predominately a result of perceived challenges when working on groups of animals as opposed to single individuals. Importantly, recent technological and statistical advances mean that some of the common challenges associated with working on a group-level can be overcome. Below we highlight some potential barriers to incorporating a social context and recommend solutions.

- **Barrier**: Increased logistical complexity or need for new experimental protocols/set-ups (e.g., larger space and time requirements). **Solution**: A social context can be incorporated into most existing protocols/set-ups by housing and testing animals in groups. Even implementing a reduced/simplified social context (i.e., smaller groups than would naturally occur) is a step toward ecological relevance.
- **Barrier**: Difficulty maintaining individual identities to measure end points over time (e.g., growth, reproduction, behavior). **Solution**: At the most basic level, visual or scan-based methods can be used to identify individuals over time (e.g., visual implant elastomer, passive integrated transponders). Recent advances in video tracking technologies even enables unmarked identification of animals in complex groups (e.g., EthoVison, TRex, ToxTrack).
- **Barrier**: Including the social environment means you must record social behaviors. **Solution**: Adding a social context does not necessitate the measurement of social behavior. Although, doing so may provide insights into the impacts of the chemical in question.
- **Barrier**: Requires a larger number of animal replicates. **Solution**: This is to some degree unavoidable, but if individual identity can be maintained during testing, animals can still be measured on an individual level, and the variability between groups can be measured and accounted for using multivariate models.
- **Barrier**: Data analysis may require more complex statistical approaches. **Solution**: The statistical techniques that may be required for group-level analyses (e.g., multivariate and complex system modeling) are becoming more common in environmental science, and there are now many general guides and free online lectures available on these procedures.

In summary, the natural social environment of many animals can be complex, which challenges our ability to extrapolate the results of laboratory studies to natural settings. Yet, it is a source of complexity that we believe can be addressed with relatively minor changes to common laboratory methodologies. This is particularly relevant for the emerging subfield of behavioral ecotoxicology. As behavioral endpoints become more established in ecotoxicology and risk assessment, we have the chance to normalize social environment as a key experimental design consideration.^6^ In many cases a single individual ***can not*** tell us the collective story; but, by routinely incorporating social context in ecotoxicological studies we can improve the predictive power of a laboratory studies to natural ecosystems.
